# Peptide optimization to improve antigen presentation and antitumor responses in sarcoma

**DOI:** 10.3389/fimmu.2026.1960095

**Published:** 2026-10-01

**Authors:** Angela Mauriello, Concetta Ragone, Beatrice Cavalluzzo, Anna Lucia Tornesello, Ottavia Clemente, Fernanda Picozzi, Michele Gallo, Salvatore Tafuto, Luigi Buonaguro, Maria Tagliamonte

**Affiliations:** 1 Innovative Immunological Models Unit, National Cancer Institute IRCCS - “Fond G. Pascale”, Naples, Italy; 2 Sarcoma and Rare Tumors Medical Oncology Unit, National Cancer Institute IRCCS - “Fond G. Pascale”, Naples, Italy; 3 Orthopedic Oncologic Surgery, National Cancer Institute IRCCS - “Fond G. Pascale”, Naples, Italy

**Keywords:** cancer vaccines, heteroclitic peptides, immunotherapy in sarcoma, improvement of antigen immunogenicity, T-cell cross-reactivity, tumor-associated antigens

## Abstract

Sarcomas are rare and heterogeneous malignancies for which therapeutic options remain limited, particularly in advanced disease. Standard treatments, including surgery, radiotherapy, chemotherapy, and approved targeted agents, provide marginal improvements in progression-free and overall survival, highlighting the need for immunotherapeutic strategies. Therapeutic cancer vaccines represent a promising strategy, but clinical efficacy has been limited by low antigen immunogenicity and an immunosuppressive tumor microenvironment (TME). Enhancing antigen presentation and T-cell receptor (TCR) engagement through heteroclitic peptide engineering may overcome immune tolerance and improve vaccine efficacy. Preliminary data from our group demonstrate that heteroclitic peptides modified at TCR-facing and anchor residues enhance antigen-specific T-cell recognition, cross-reactivity with wild-type epitopes, and tumor control in preclinical models. In this study, we aim to design and validate optimized heteroclitic epitopes derived from sarcoma-associated TAAs. We developed an integrated computational and experimental workflow to identify and optimize HLA-A*02:01-restricted epitopes derived from sarcoma-associated TAAs. Candidate wild-type (WT) peptides were selected using NetMHCpan 4.1 based on predicted HLA binding affinity and subsequently engineered through systematic amino acid substitutions to generate heteroclitic variants. Optimized peptides were evaluated by structural modeling and molecular docking, followed by experimental *in vitro* validation using T2-cell HLA stabilization assays. Their immunogenicity was assessed ex vivo in peripheral blood mononuclear cells (PBMCs) isolated from HLA-A*02:01-positive sarcoma patients using IFN-γ ELISpot and peptide–HLA dextramer analyses. The sequential optimization strategy generated heteroclitic variants with markedly improved predicted HLA-A*02:01 binding for most target proteins. Structural modeling indicated preservation of the peptide backbone and TCR-accessible surface for the majority of selected variants. These predictions were supported experimentally: several heteroclitic peptides enhanced HLA-A*02:01 stabilization and prolonged peptide–MHC complex persistence in T2 cells. Ex vivo, optimized peptides induced stronger antigen-specific IFN-γ responses than their wild-type counterparts, with significant increases for SET, CAGE, PTPN1 and MAGE-A3, while multimer staining demonstrated recognition of heteroclitic peptide–HLA complexes by wild-type-primed CD8^+^ T cells. These findings establish a transferable workflow for engineering more immunogenic, yet antigenically faithful, HLA-A*02:01-restricted epitopes. Rational heteroclitic optimization may therefore provide a useful platform for the development of next-generation therapeutic vaccines for sarcoma and other tumors with low intrinsic immunogenicity.

## 1 Introduction

Sarcomas represent a highly heterogeneous group of mesenchymal malignancies, characterized by their aggressive course and poor response to treatment. They account for less than 1% of all adult solid malignancies and approximately 15% of pediatric cancers (1, 2). According to current classifications, sarcomas are broadly categorized into bone sarcomas, visceral sarcomas, most notably gastrointestinal stromal tumors (GIST) and soft tissue sarcomas (STS), which arise from connective, subcutaneous, and other soft tissues including adipose tissue, muscle, nerves, fibrous tissue, and blood vessels (3). The World Health Organization recognizes nearly 100 histological sarcoma subtypes, more than 50 of which belong to STSs, which represent the majority of diagnosed cases, whereas bone sarcomas account for approximately 10% (4). Despite the identification of several risk factors, including age, prior radiation exposure, previous malignancies, and genetic predisposition, the etiology of most sarcomas remains poorly understood (5, 6).

Standard treatment strategies include surgery, radiotherapy, and chemotherapy (7). Surgery remains the first-choice treatment for localized disease, aiming at local tumor control, while chemotherapy is the main therapeutic option in the metastatic setting. In localized disease, chemotherapy may be administered in the neoadjuvant setting to reduce tumor burden or as adjuvant therapy in high-risk cases to decrease the likelihood of local or distant relapse. More recently, targeted agents such as pazopanib, trabectedin, and eribulin have been approved for unresectable or metastatic STS. However, these therapies confer only modest benefits in progression-free survival (PFS) and overall survival (OS) and are rarely curative (8–11). Collectively, these limitations highlight the urgent need for alternative therapeutic strategies capable of improving long-term outcomes for sarcoma patients.

In this context, immunotherapy, and in particular therapeutic cancer vaccination, represents a promising approach. Cancer vaccines aim to induce *de novo* tumor-specific immune responses or to amplify pre-existing antitumor immunity. A major challenge in vaccine development is the identification of suitable tumor antigens capable of eliciting robust and durable T-cell responses. Ideal vaccine targets include tumor-associated antigens (TAAs) and tumor-specific antigens (TSAs), selectively or preferentially expressed by cancer cells (12, 13).

Several TAAs have been identified in sarcomas, including cancer–testis antigens (CTAs), which are normally restricted to germ cells but aberrantly expressed in tumors. Among these, NY-ESO-1, MAGE, PRAME, BAG, and CAGE are frequently overexpressed in sarcomas (14–17). NY-ESO-1 expression is particularly prominent in synovial sarcoma, where it is detected in up to 85% of cases, as well as in round-cell myxoid liposarcoma (18). In addition, PRDX2 and DDX39A have been functionally implicated in Ewing sarcoma and osteosarcoma, respectively, whereas PRDX5 and PFETIN have been associated with gastrointestinal stromal tumor biology and clinical outcome. SET overexpression has been demonstrated in alveolar soft-part sarcoma, while PTPN1, SCGF and members of the BAG protein family have been linked to signaling, immune regulation or disease progression in selected sarcoma histotypes (19–25). Moreover, specific chromosomal translocations characteristic of certain sarcoma subtypes generate fusion proteins that represent attractive tumor antigens, such as the t(X;18)(p11;q11) translocation in synovial sarcoma (26, 27). Despite these advances, clinical trials evaluating vaccines targeting TAAs or fusion-derived antigens have yielded disappointing results, with limited clinical benefit (28–30).

The suboptimal efficacy of sarcoma vaccines can be attributed to at least two major factors: the immunosuppressive tumor microenvironment (TME) and the intrinsic low immunogenicity of the target antigens included in vaccine formulations. Strategies aimed at modulating the TME such as combining vaccines with anti-angiogenic or immunomodulatory agents, have shown promise in other tumor types (31–35), but their application in sarcomas remains challenging due to the still incomplete characterization of the sarcoma immune landscape.

With regard to antigen selection, most sarcoma associated antigens are wild-type self-proteins overexpressed by tumor cells and therefore subject to central and peripheral immune tolerance. To overcome this limitation and enhance antigen immunogenicity, peptide engineering approaches could be used to increase their affinity and binding to the presenting MHC-I (36–39). One strategy to overcome this limitation is the development of heteroclitic peptides, in which selected amino acid substitutions are introduced to improve peptide-MHC interactions while preserving the structural determinants required for T-cell receptor (TCR) recognition. Unlike native epitopes, heteroclitic peptides are designed to enhance the stability of peptide-MHC complexes, thereby increasing the magnitude and duration of antigen presentation and promoting stronger T-cell activation. In previous preclinical *in vivo* studies we have demonstrated that rational modification of canonical MHC anchor residues can significantly enhance peptide immunogenicity without abolishing cross-recognition of the corresponding wild-type epitope, making heteroclitic peptides attractive candidates for therapeutic cancer vaccines (40–42). Low-affinity interactions between the TCR and peptide–MHC complexes are known to promote TCR cross-reactivity, providing a mechanistic basis for the effectiveness of heteroclitic optimization strategies.

Alternative heteroclitic approaches focusing on modifications of TCR contact residues have also demonstrated enhanced immunogenicity. Preliminary results from our group showed that heteroclitic modification of TCR-facing residues within the melanoma-associated Trp2 antigen significantly improved tumor growth control *in vivo*. Similarly, modification of TCR facing amino acids, particularly at position P4, of the HPV E7 epitope enhanced recognition of the wild-type peptide by peripheral blood mononuclear cells (PBMCs). Moreover, heteroclitic peptides combining substitutions at both TCR-facing and anchor residues elicited even stronger immune responses, cross-reacting with the parental wild-type peptide. CTLs elicited by these heteroclitic peptides displayed potent lytic activity against target cells presenting the wild-type epitope and controlled tumor growth *in vivo* (40–42).

Against this background, we sought to develop a rational strategy for improving the immunogenicity of HLA-A*02:01-restricted epitopes derived from sarcoma-associated target proteins.

We combined literature guided target selection, NetMHCpan-based epitope prediction, motif-guided optimization of HLA anchor residues, systematic refinement of the TCR-facing P5 position, structural modeling, followed by *in vitro* and *ex vivo* immunological validation. The resulting peptide panel was evaluated for HLA-A*02:01 binding and complex stability in T2 cells and for functional immunogenicity and cross-recognition in PBMCs from HLA-A*02:01-positive sarcoma patients (**Figure 1**).

**Figure 1:**
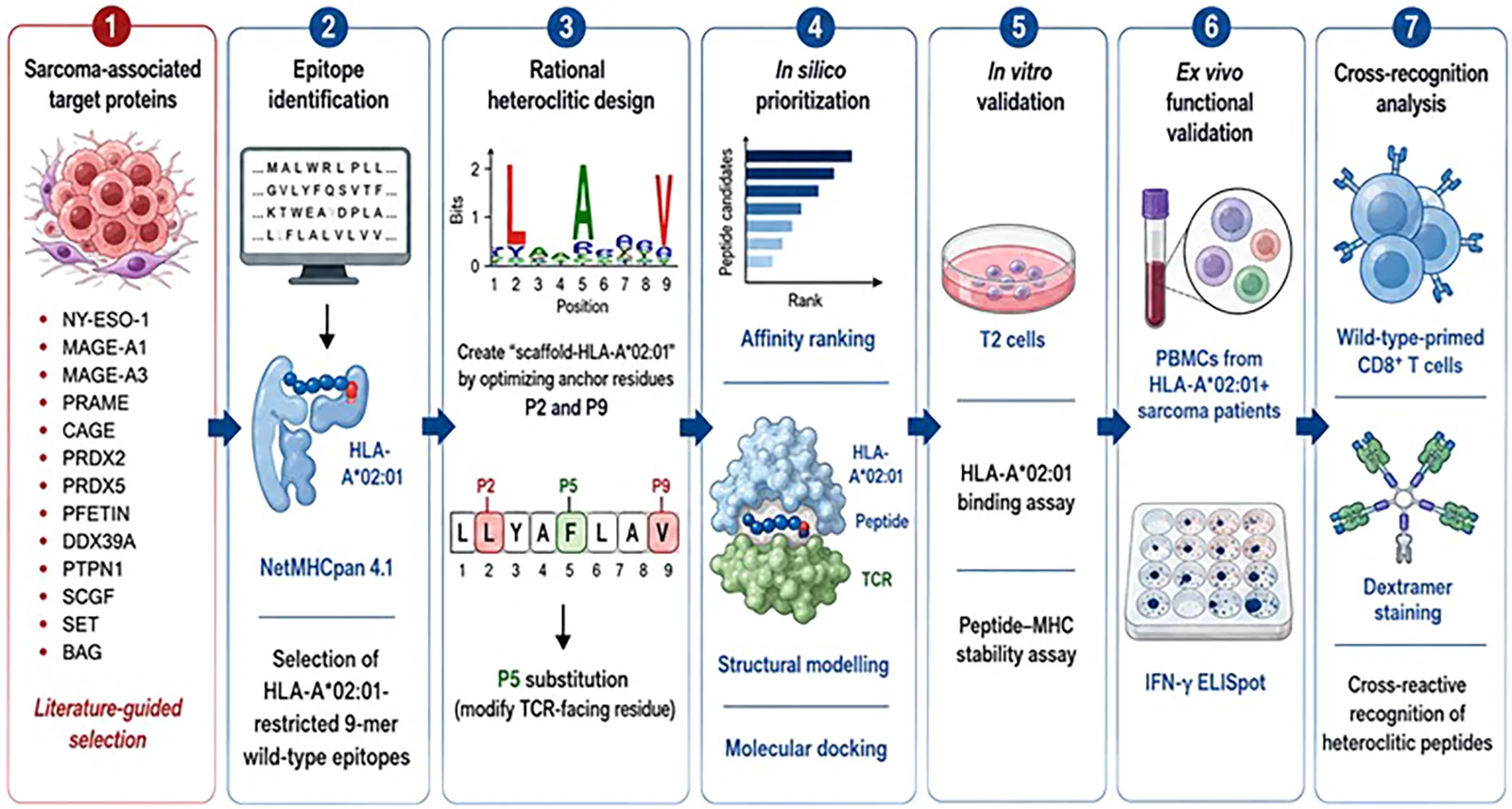
Integrated workflow for the rational design and validation of heteroclitic peptides targeting sarcoma-associated antigens. Sarcoma-associated target proteins were selected on the basis of published evidence of overexpression, immunogenicity or functional involvement in selected sarcoma histotypes. Full-length protein sequences were analyzed using NetMHCpan 4.1 to identify HLA-A*02:01-restricted 9-mer wild-type epitopes. Candidate peptides were then subjected to a sequential engineering strategy consisting of motif-guided optimization of the HLA anchor residues at positions P2 and P9, followed by systematic substitution of the predominantly TCR-facing residue at P5. Wild-type, scaffold-HLA-A*02:01 and heteroclitic variants were re-ranked according to predicted binding affinity and further prioritized by structural modeling and molecular docking. Selected peptide pairs were experimentally evaluated in TAP-deficient T2 cells for HLA-A*02:01 binding and peptide–MHC complex stability. Their functional immunogenicity was assessed ex vivo in PBMCs from HLA-A*02:01-positive sarcoma patients by IFN-γ ELISpot, while dextramer staining was used to investigate recognition of heteroclitic peptide–HLA complexes by wild-type-primed CD8^+^ T cells. This integrated workflow identified optimized heteroclitic peptides with enhanced HLA-A*02:01 binding, increased immunogenicity and preserved cross-recognition of the corresponding native epitopes.

## 2 Materials and methods

### 2.1 Selection of sarcoma-associated target proteins and identification of parental epitopes

Thirteen target proteins were selected through a literature, based review of antigens reported to be overexpressed and/or immunogenic in sarcoma. The panel comprised NY-ESO-1, MAGE-A1, MAGE-A3, PRAME, CAGE, PRDX2, PRDX5, PFETIN, DDX39A, PTPN1, SCGF, SET and BAG. Full-length protein sequences were retrieved from the UniProt Knowledgebase (https://www.uniprot.org; accessed 28 October 2025). Each sequence was screened using NetMHCpan4.1 algorithm (http://www.cbs.dtu.dk/services/NetMHCpan/) to identify HLA-A*02:01 restricted 9-mer peptides. Candidate epitopes were ranked according to predicted binding affinity and percentile rank and classified as strong (SB) or weak binders (WB) using the default NetMHCpan thresholds (the lower the value, the higher the affinity). One parental wild-type epitope was selected from each target protein on the basis of predicted HLA-A*02:01 binding and the overall suitability of the sequence for subsequent engineering.

### 2.2 Rational design and in silico prioritization of heteroclitic peptides

Heteroclitic peptides were generated using a sequential, motif guided optimization strategy. First, amino-acid preferences at the principal HLA-A*02:01 anchor positions were derived from the sequence logo provided by NetMHCpan 4.1. Non-preferred residues at P2 and/or P9 were replaced with residues enriched in the HLA-A*02:01 motif to generate an intermediate scaffold-HLA-A*02:01 sequence, while the remaining positions were initially left unchanged.

In a second step, the predominantly TCR-facing residue at P5 was systematically replaced with each of the 20 naturally occurring amino acids. All scaffold-HLA-A*02:01 and P5-substituted sequences were re-analyzed with NetMHCpan 4.1. Predicted binding affinity, percentile rank and binder classification were compared with those of the parental wild-type peptide. Variants showing the strongest predicted improvement, while retaining a sequence compatible with structural and functional evaluation, were prioritized for further analysis.

### 2.3 Structural modeling and molecular docking

Structural modeling was performed as a comparative, structure-guided analysis to assess the effects of anchor and P5 substitutions on peptide conformation within the HLA-A02:01 binding groove and on the orientation of solvent-exposed, potentially TCR-facing residues. Wild-type, scaffold-HLA-A02:01 and selected heteroclitic sequences were modelled using PyMOL (version 1.8.6.2) and Molsoft ICM (version 3.8-7d). The experimentally determined HLA-A*02:01–peptide–TCR complex 1AO7 was retrieved from the RCSB Protein Data Bank and used as a common structural framework for comparative modeling. The original TAX peptide was computationally replaced with each selected 9-mer sequence.

Models were comparatively inspected for preservation of the overall peptide backbone, positioning within the HLA-A*02:01 binding groove, and orientation of solvent-exposed side chains potentially involved in TCR engagement. The modeling was not intended to provide *de novo* structural predictions or quantitative estimates of peptide–HLA or peptide–TCR binding affinity, but rather to visualize the structural consequences of the designed substitutions within a consistent experimentally determined peptide–HLA–TCR framework.

### 2.4 Peptide synthesis

Peptide synthesis was performed using a fully automated parallel peptide synthesizer (Syro I, Biotage), employing the conventional Fmoc (9-fluorenylmethoxycarbonyl)/OBut solid-phase peptide synthesis (SPPS) strategy (43). Wang resin preloaded with the first amino acid was employed as the solid support to afford peptides with a C-terminal carboxylic acid. Nα Fmoc amino acids (4.0 eq., 0.5 M) and the coupling reagent 2-(1H-Benzotriazole-1-yl)-1,1,3,3-tetramethylaminium hexafluorophosphate (HBTU, 3.9 eq., 0.48M) were used with N,N-diisopropylethylamine (DIPEA, 7.8eq., 2.0M in 1-metil-2-pirrolidone, N-metil-2-pirrolidone (NMP)) in N,N-dimetilformammide (DMF). After each coupling step, the resin was washed three times with DMF, and the Fmoc protecting group was removed using 20% piperidine in DMF. Once peptide elongation and final Nα Fmoc deprotection were completed, the resin was washed successively six times with DMF and two times with dichloromethane (DCM), then dried thoroughly. Peptidyl-resins were cleaved from the solid support by treatment with trifluoroacetic acid (TFA), triisopropylsilane (TIS) and ddH_2_O (95:2.5:2.5) for 3 hours. The cleavage cocktail was collected by filtration and the peptides were isolated by precipitation in could diethyl ether. The final crude compounds were purified by reverse phase high-performance liquid chromatography (RP-HPLC) using a C18 column (Aeris 250x21.2, 5 μm Phenomenex), eluted with H_3_O containing 0.1% TFA (solvent A) and CH_3_CN containing 0.1% TFA (solvent B) using a linear gradient from 5 to 65% B over 20 min at 10 mL/min. Molecular weight was confirmed by liquid chromatography-time-of-flight mass spectrometry (LC-TOF-MS, Agilent 6230). Analytical HPLC analysis were conducted using a C18 Aeris column (5 μm, 150x4.6 mm) eluted with H_3_O containing 0.1% formic acid (solvent A) and CH_3_CN containing 0.1% formic acid (solvent B) using a linear gradient from 5 to 65% B over 15 min at 0.5 mL/min. The final purified compounds were obtained in good yield (85%) and with high purity grade (>95%).

All materials were obtained from commercial suppliers, amino acids and preloaded wang resin were purchased from Merck; TFA, DMF, NMP, Piperidine DIPEA, formic acid and TIS were obtained from Sigma-Aldrich. All solvents were of reagent grade and used without further purification.

### 2.5 T2-cell HLA-A*02:01 binding and stability assays

Peptide binding affinity to HLA-A*02:01 molecule and BFA decay assays were performed for each candidate peptide. Human TAP-deficient T2 cell line (174xCEM.T2;ATCC CRL 1992™) was purchased from American Type Culture Collection (ATCC; https://www.atcc.org/ (accessed on 3 March 2021)) and cultured in Iscove’s modified Dulbecco’s medium (IMDM; Gibco Life Technologies, Thermo Fisher Scientific Inc., Waltham, MA,USA) containing 25 mM HEPES and 2 mM L-glut, supplemented with 20% fetal bovineserum (FBS; Capricorn Scientific GmbH, Ebsdorfergrund, Germany), 100 IU/mL penicillin and 100 mg/mL streptomycin (Gibco Life Technologies). Cells were maintained at 37 °C in a humidified incubator with 5% CO2. Briefly, T2 cell lines were seeded at 3.5 x10^5^ cells per well in 24-well plates and incubated for 16 h at 27 °C with peptides (final concentrations: 5, 10, 20, 50, and 100 uM) in IMDM serum-free medium. The next day, cells were incubated for an additional 2 h at 37 °C. Following incubation, cells were harvested and centrifuged at 200 x g for 5 min. Subsequently, cells were washed twice with phosphate-buffered saline (1X PBS; Gibco Life Technologies) and stained with R-PE conjugated anti-human HLA-A2 monoclonal antibody (cat. 343306; BioLegend, San Diego, CA, USA), for 30 min at 4 °C, and analyzed with the Attune™ NxT flow cytometer (Thermo Fisher Scientific). Mouse H-2Kb-specific OVA SIINFEKL peptide was used as a negative control, and T2 cells without any added peptide were used as a background control. HLA-A*02:01 MUC1 LLLLTVLTV peptide was used as a positive control. A fluorescence index (FI) was calculated using the following formula: FI = mean fluorescence intensity (MFI) sample MFI background/MFI background, where MFI background represents the value without peptide. A value of FI > 0.5 was set as a threshold to indicate peptides with affinity for the HLA-A*02:01 molecule. For the brefeldin A decay assay, T2 cells were incubated with peptides (50 mM) as described above, washed, and treated with 1X BFA (brefeldin A solution; cat. 420601; BioLegend) in IMDM serum-free medium, for 1 h at 37 °C. Cells were harvested every two hours (T0, T2, T4, T6, T8), washed with phosphate-buffered saline (1X PBS; Gibco Life Technologies), stained with anti-HLA-A*0201 fluorescent monoclonal antibody (cat. 343306; BioLegend), and analyzed by flow cytometry. The stability of each peptide bound to HLA-A2 was measured as the DC50 value, which was defined as an estimate of the time required for a 50% reduction in the MFI value at time 0. The DC50 value was calculated according to the following formula: MFI at indicated time points/MFI at time 0 _ 100. All the experiments were performed in triplicate.

### 2.6 Patient enrolment, sample collection and HLA typing

The study was approved by the Institutional Ethics Committee (Protocol n. 58/22 OSS) and was conducted in accordance with the Declaration of Helsinki. Written informed consent was obtained from all participants before sample collection. A total of 70 patients with different sarcoma histotypes were enrolled and screened for HLA-A02:01 expression. Peripheral blood mononuclear cells were collected from all participants and HLA-A2 surface expression was assessed by flow cytometry using a PE-conjugated anti-human HLA-A2 monoclonal antibody (clone BB7.2, BioLegend, San Diego, CA, USA), according to the manufacturer’s instructions. Only samples from HLA-A*02:01-positive subjects were selected for the immunological analyses described in this study. In particular, among the total subjects enrolled, 22 patients (31.4%) were HLA-A02:01 positive and 48 (68.6%) were HLA-A02:01 negative (**Supplementary Table S1**). Peripheral blood mononuclear cells (PBMCs) were isolated from peripheral blood by density-gradient centrifugation by Ficoll-Hypaque and cryopreserved in 10% of DMSO and stored in liquid nitrogen until use.

### 2.7 IFN-γ ELISpot assay

Antigen specific T-cell responses were assessed in PBMCs from HLA-A*02:01 positive sarcoma patients. 2×10^6^ PBMCs were stimulated with individual heteroclitic peptides at 10 µg/mL and cultured for 14 days in 100 µL RPMI 1640 medium (Capricorn Scientific GmbH). On day three, 10 U/mL IL-2 was added to each well. On day five, half of the volume of medium was replaced with fresh medium containing IL-2 at a final concentration of 10 U/mL. On day seven, PBMCs were restimulated with each peptide. On day 14, cells were harvested for IFN-γ ELISpot assay. The plates were read with an AID EliSpot Reader Systems (AID GmbH, Strassberg, Germany). Determinations from triplicate tests were averaged. Paired peptides (WT and HET) were added at a final concentration of 10 μg/mL to 2×10^5^ PBMCs per well in 100 μL RPMI 1640 medium (Capricorn Scientific GmbH). PBMCs were cultured at 37 °C in a humidified incubator with 5% CO2 for 20 hours. Stimulation with 10 μg/mL Phytohemagglutinin (PHA-K; Capricorn Scientific GmbH) was used as positive control, PBMCs without added peptides were used as the negative control, RPMI 1640 medium (Capricorn Scientific GmbH) was used as background control. The plates were read with an AID EliSpot Reader Systems (AID GmbH, Strassberg, Germany). Determinations from triplicate tests were averaged.

Data were analyzed by subtracting the mean number of spots in the wells with cells and medium only from the mean counts of spots in wells with cells and antigen. Spot forming units (SFU) were calculated as the frequency per 10^6^ PBMCs.

### 2.8 Peptide-HLA multimer staining and flow-cytometric analysis

Peptide-HLA complexes were generated by incubating HLA-A*02:01 monomers (100 µg/mL) with 100 µM peptide for 30 min in PBS at room temperature. Complexes were centrifuged at 3,300 × g for 5 min to remove aggregates. D-biotin was added to a final concentration of 25 µM to block unoccupied streptavidin-binding sites. Multimers were stored at −20 °C in PBS containing 5% glycerol and 0.5% BSA.

To assess cross-recognition, PBMCs were stimulated with individual wild-type peptides (10 µg/mL) for 7 days and then incubated with HLA-A*02:01 multimers loaded with the wild-type or corresponding heteroclitic peptide for 15 min at 37 °C. Cells were stained with CD3- Super Bright 436 (1:40; eBioscience, 62-0037-42), CD8- Pe-Cy7 (1:100; eBioscience, 25-0088-42) and LIVE/DEAD Fixable Near-IR (1:1,000; Invitrogen, L10119) for 30 min on ice. Samples were acquired on an Attune NxT Acoustic Focusing Cytometer and analyzed after sequential gating on singlets, live lymphocytes and CD3^+^CD8^+^ T cells. Wild-type multimer-positive, heteroclitic multimer-positive and double-positive populations were quantified as percentages of CD8^+^ T cells. A representative flow-cytometry gating strategy, including the sequential identification of lymphocytes, singlets, viable CD3^+^CD8^+^ T cells, and WT- and/or HET-dextramer-positive populations, is shown in **Supplementary Figure S1**.

### 2.9 Statistical analysis

Comparison between individual data points were performed with the unpaired two-sided Student’s t-test and ANOVA, as appropriate. Normally distributed data were represented as mean ± S.E.M. Two way ANOVA and Bonferroni *post-hoc* analysis were used to examine the significance of differences among groups. All P values were two tailed and considered significant if less than 0.05.

## 3 Results

### 3.1 Identification of HLA-A*02:01-restricted epitopes from sarcoma-associated target proteins

To identify parental epitopes suitable for subsequent heteroclitic modification, the full-length amino-acid sequences of 13 sarcoma-associated target proteins were retrieved from UniProt and analyzed using NetMHCpan 4.1 for the prediction of HLA-A*02:01-restricted 9-mer peptides (**Figure 2A**). The selected panel included NY-ESO-1, PRDX5, CAGE, MAGE-A1, MAGE-A3, PTPN1, SCGF, SET, PRDX2, PFETIN, DDX39A, PRAME and BAG.

**Figure 2:**
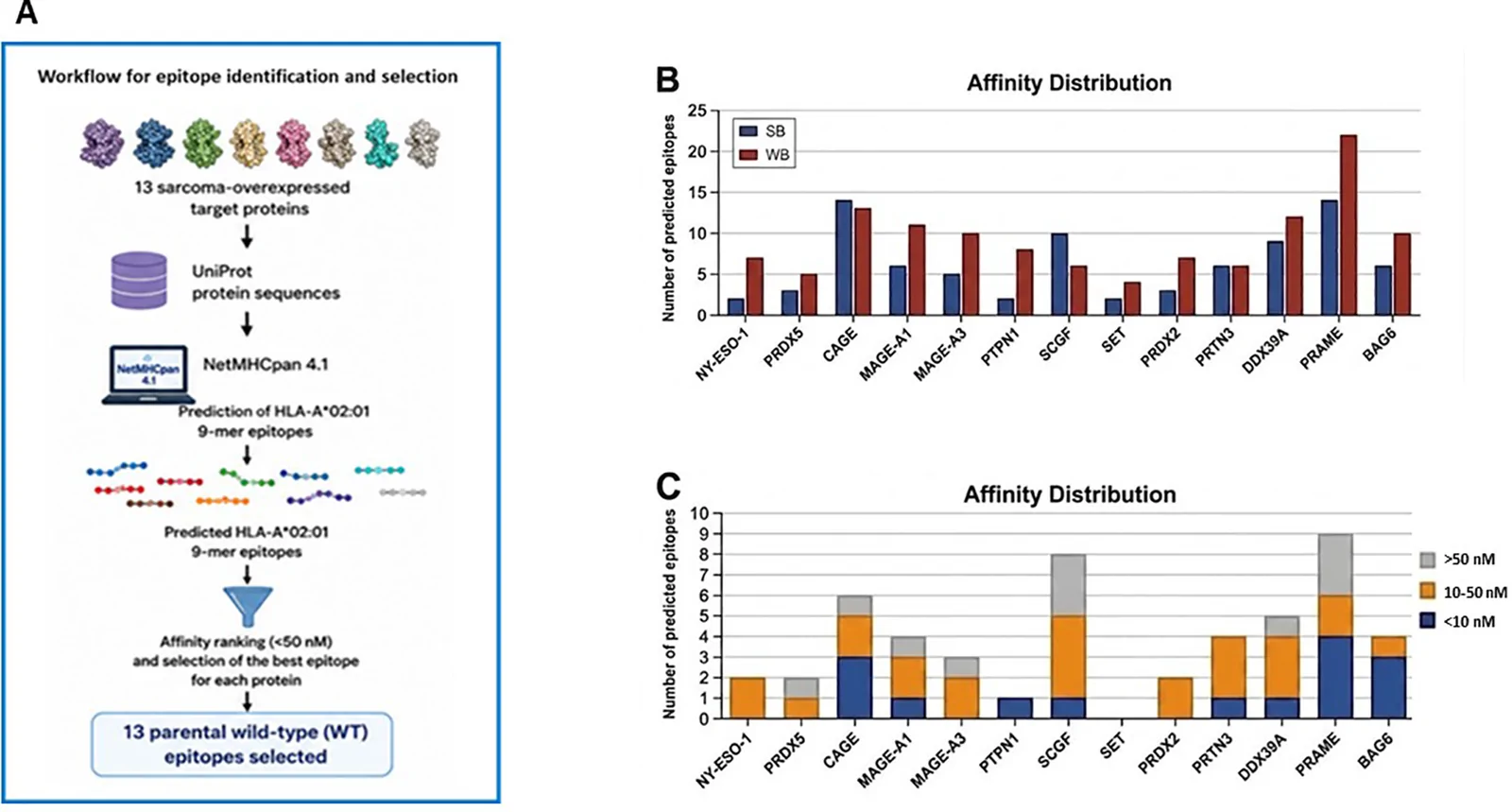
Distribution of predicted HLA-A*02:01-restricted epitopes across sarcoma-associated target proteins. Full-length protein sequences were analyzed using NetMHCpan 4.1 to identify candidate 9-mer peptides predicted to bind HLA-A*02:01. Bars indicate the number of predicted epitopes identified for each target protein and are stratified according to predicted binding affinity: >50 nM, 10–50 nM and <10 nM. Lower affinity values correspond to stronger predicted HLA-A*02:01 binding.

The initial screening revealed marked differences in the number of predicted HLA-A*02:01 binders across the analyzed proteins (**Figure 2B**). PRAME generated the largest repertoire of predicted peptides, with 14 strong binders and 22 weak binders, followed by CAGE, with 14 strong and 13 weak binders. A relatively high number of predicted binders was also identified for DDX39A, which yielded 9 strong and 12 weak binders, and for SCGF, with 10 strong and 6 weak binders. MAGE-A1 and MAGE-A3 generated 6 and 5 strong binders and 11 and 10 weak binders, respectively, whereas BAG yielded 6 strong and 10 weak binders. Lower numbers of predicted peptides were observed for NY-ESO-1, PRDX5, PTPN1, SET and PRDX2. These results indicated that the density of potential HLA-A*02:01-restricted epitopes varied considerably among the selected sarcoma-associated proteins.

Candidate peptides were subsequently stratified according to their predicted binding affinity into three intervals: >50 nM, 10–50 nM and <10 nM, with lower values indicating stronger predicted HLA-A*02:01 binding (**Figure 2C**). Following this affinity-based selection, a total of 50 candidate wild-type epitopes were retained across the protein panel. PRAME contained the highest number of selected peptides (n = 9), including four peptides with predicted affinities above 50 nM, two within the 10–50 nM range and three below 10 nM. SCGF yielded eight candidates, including three peptides with predicted affinities below 10 nM, while CAGE generated six candidates distributed across all three affinity categories. Five candidates were retained for DDX39A, four each for MAGE-A1, PFETIN and BAG, three for MAGE-A3, two each for NY-ESO-1, PRDX2 and PRDX5, and one for PTPN1.

The relative distribution of affinity classes also differed among proteins. Several targets, including PRDX5, MAGE-A1, MAGE-A3, SCGF, DDX39A and PRAME, generated at least one peptide with a predicted affinity below 10 nM. By contrast, the candidates identified for NY-ESO-1, PFETIN and BAG were predominantly distributed within the 10–50 nM or >50 nM intervals. No SET-derived peptide fulfilled the predefined affinity threshold. Nevertheless, because SET was considered a biologically relevant sarcoma-associated target, its highest-ranking 9-mer peptide was retained and included in the subsequent optimization workflow.

For each target protein, one parental wild-type epitope was ultimately selected by integrating predicted HLA-A*02:01 binding affinity, binding rank and suitability for subsequent sequence engineering. This process generated a final panel of 13 parental wild-type peptides, one for each sarcoma-associated target protein, which were advanced to motif-guided anchor optimization and systematic P5 substitution.

### 3.2 Sequential peptide engineering generates optimized HLA-A*02:01-binding heteroclitic variants

One parental HLA-A*02:01 restricted wild-type epitope from each target protein was subjected to a two-step engineering procedure. First, an HLA optimized scaffold was generated by replacing non-preferred residues at P2 and/or P9 according to the HLA-A*02:01 sequence motif.

Sequence Logo analysis of HLA-A*02:01-binding motifs confirmed the expected preference for canonical anchor residues, with a strong enrichment of hydrophobic amino acids, particularly Leu at position 2 and Val/Leu at the C-terminal anchor position (P9), consistent with the established HLA-A*02:01 binding motif (**Figure 3A**). These positional constraints guided the rational selection of amino acid substitutions aimed at maximizing peptide-MHC stability while preserving the T-cell receptor-facing residues.

**Figure 3:**
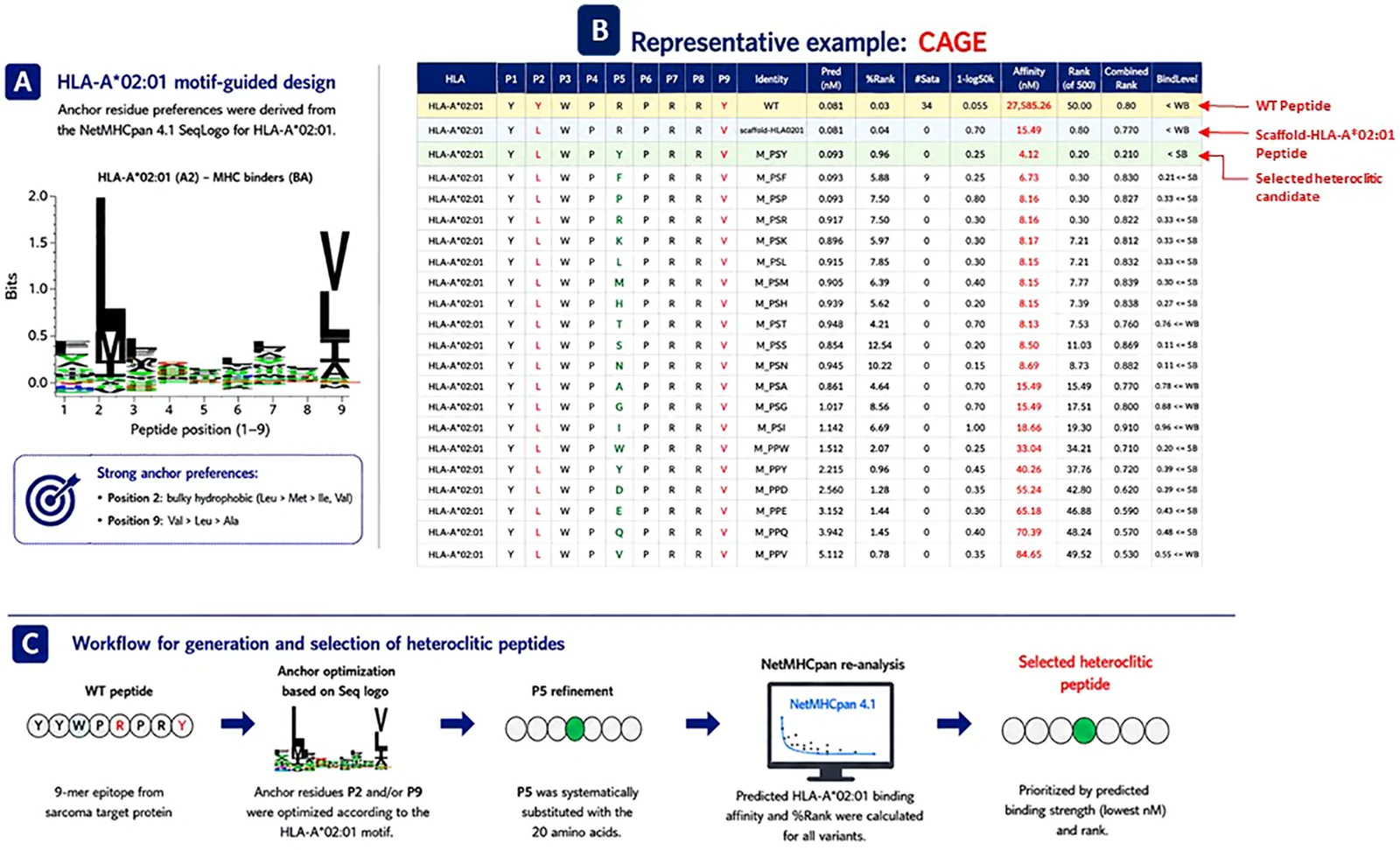
Motif-guided sequential engineering and selection of HLA-A*02:01-restricted heteroclitic peptides.**(A)** Sequence logo of HLA-A*02:01-binding peptides generated using NetMHCpan 4.1, showing preferential amino acid usage at the principal anchor positions P2 and P9. **(B)** Representative optimization of the CAGE-derived wild-type epitope. Anchor-residue substitution generated an HLA-optimized scaffold, followed by systematic amino acid replacement at the TCR-facing P5 position. Variants were ranked according to predicted HLA-A*02:01 binding affinity and percentile rank. **(C)** Schematic representation of the sequential peptide-engineering workflow, including wild-type epitope selection, motif-guided anchor optimization, P5 refinement, NetMHCpan re-analysis and prioritization of the final heteroclitic candidates.

In the second step, the predominantly TCR-facing residue at P5 was systematically substituted with each of the 20 naturally occurring amino acids. The resulting variants were re-analyzed with NetMHCpan 4.1, allowing HLA anchor optimization to be combined with controlled refinement of the TCR-accessible surface.

The CAGE-derived epitope provides a representative example of this workflow (**Figure 3B**). The parental WT peptide YYWPRPRRY showed very low predicted HLA-A*02:01 binding, with an estimated affinity of 27,585.26 nM. Motif-guided replacement of the anchor residues generated the scaffold peptide YLWPRPRRV, resulting in a marked improvement in predicted affinity to 15.49 nM. Subsequent substitution of P5 with tyrosine generated the final heteroclitic peptide YLWPYPRRV, which further improved the predicted affinity to 4.12 nM. Thus, sequential optimization converted the CAGE-derived WT sequence from a very weakly predicted binder into a high-affinity HLA-A*02:01 candidate (SB).

The same design strategy was applied across the remaining target proteins selected. In several cases, the scaffold alone substantially improved the predicted HLA-A*02:01 affinity, whereas additional P5 substitution provided a further gain. Representative examples included CAGE, BAG, MAGE A1, MAGE A3, SET, PFETIN, NY-ESO-1 (**Figure 4**). Conversely, when modification did not improve prediction, as observed for the NY-ESO-1 derived peptide, only the WT sequence was retained for subsequent evaluation.

**Figure 4:**
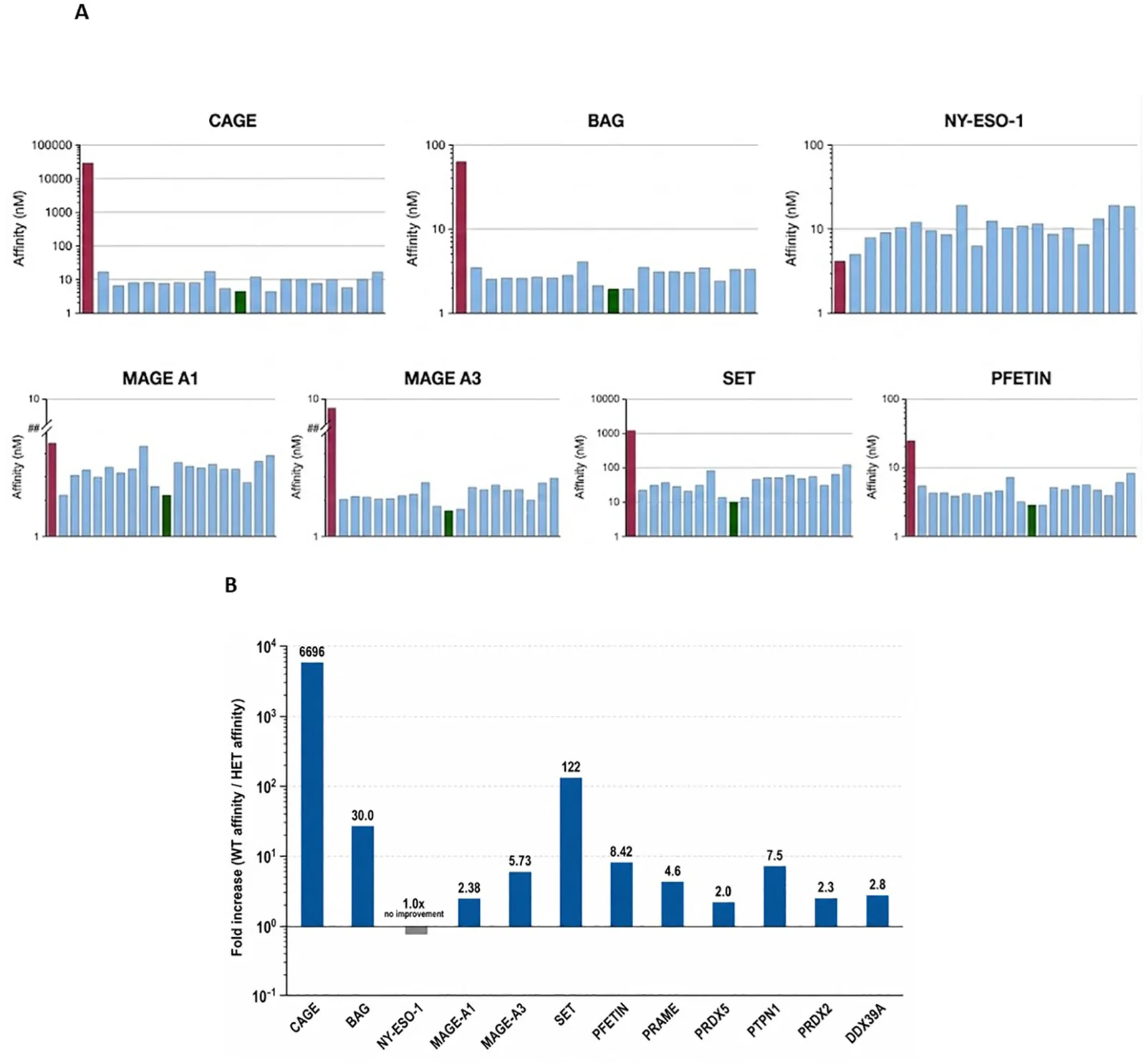
**(A)** Predicted HLA-A*02:01 binding-affinity profiles of wild-type and heteroclitic peptide variants. Predicted affinities of parental wild-type peptides, scaffold-HLA-A*02:01 sequences and P5-substituted variants were calculated using NetMHCpan 4.1. Lower values indicate stronger predicted binding. Red bars denote parental peptides (WT), light-blue bars all engineered variants and green bars the candidates selected for experimental validation (HET). **(B)** Fold increase in predicted HLA-A*02:01 binding affinity of selected heteroclitic peptides relative to their parental wild-type epitopes. Fold increase was calculated as the ratio between the predicted affinity of each parental wild-type (WT) peptide and that of the corresponding selected heteroclitic (HET) variant. Because lower nM values indicate stronger predicted HLA-A*02:01 binding, higher WT/HET ratios reflect greater improvement after heteroclitic optimization.

Overall, the motif-guided scaffold followed by P5 refinement generated a panel of optimized peptide candidates with improved predicted HLA-A*02:01 binding compared with their parental sequences (**Supplementary Table S2**). The selected WT/HET pairs were subsequently advanced to structural modeling and experimental immunological validation.

### 3.3 In silico affinity ranking identifies heteroclitic variants with enhanced predicted HLA-A*02:01 binding

The greatest improvement was observed for the CAGE-derived epitope. The wild-type peptide YYWPRPRRY showed very weak predicted binding, with an affinity of 27,585.26 nM. Anchor optimization generated the scaffold-HLA-A*02:01 sequence YLWPRPRRV, with an affinity of 15.49 nM, while subsequent substitution of P5 with tyrosine produced YLWPYPRRV, with a predicted affinity of 4.12 nM. This corresponded to an approximately 6,700-fold improvement relative to the wild-type peptide.

A substantial improvement was also observed for SET. The parental peptide HIDEVQNEI had a predicted affinity of 1,185 nM, whereas the selected tryptophan-substituted variant HLDEWQNEV reached 9.69 nM, corresponding to an approximately 122-fold improvement.

For BAG, the wild-type peptide KILEEIDTL displayed a predicted affinity of 62.41 nM. Anchor optimization generated KLLEEIDTV, with an affinity of 3.39 nM, while the selected P5-substituted variant reached a predicted affinity of approximately 2 nM, corresponding to an approximately 30-fold improvement relative to the parental sequence.

To enable a direct comparison of the effect of heteroclitic optimization across the complete peptide panel, the fold increase in predicted HLA-A*02:01 binding was calculated as the ratio between the affinity of each parental WT peptide and that of the corresponding selected HET variant (**Figure 4B**). This analysis confirmed that the greatest improvements were achieved for CAGE, SET and BAG, whereas the remaining peptide pairs showed more moderate gains.

More modest improvements were observed for PFETIN, MAGE-A1, MAGE-A3, PRAME, PRDX5, PTPN1, PRDX2 and DDX39A. Although the magnitude of the gain was lower than that observed for CAGE, SET and BAG, heteroclitic optimization generally improved the predicted HLA-A*02:01 binding affinity relative to the corresponding parental sequences.

In contrast, optimization of the NY-ESO-1-derived peptide did not produce a meaningful improvement. The wild-type peptide SLLMWITQC showed a predicted affinity of 4.0 nM, whereas the scaffold-HLA-A*02:01 sequence SLLMWITQV displayed a slightly weaker predicted affinity of 4.8 nM. Although several P5-substituted variants retained high predicted binding, none clearly outperformed the parental sequence. The wild-type NY-ESO-1 peptide was therefore retained for subsequent evaluation.

### 3.4 Comparative structural modeling indicates preservation of peptide conformation and TCR-accessible surfaces modeling

To assess whether the substitutions introduced during heteroclitic optimization affected the structural features required for immune recognition, the conformations of the WT, scaffold-HLA-A*0201 and final heteroclitic peptides were compared within the HLA-A*02:01 TCR complex.

Comparative structural modeling indicated that most selected heteroclitic variants preserved an overall peptide backbone arrangement similar to that of the corresponding WT epitopes within the common structural framework. modeling. In particular, the peptide backbone remained similarly positioned within the HLA-A*02:01 binding groove, while the side chains predicted to interact with the TCR maintained a comparable orientation.

Representative structurally conserved peptide sets included variants derived from SET, CAGE, MAGE-A1, MAGE-A3, PTPN1, DDX39A, PRDX2 and SCGF. For SET, CAGE, MAGE-A1, MAGE-A3 and PTPN1, comparison of the three sequentially generated structures WT, scaffold-HLA-A*0201 and P5 substituted heteroclitic peptide, showed that anchor optimization did not substantially alter the global peptide architecture. The additional substitution at P5 modified the chemical properties of the solvent-exposed side chain while preserving its orientation toward the putative TCR-contact surface.

Overall, 8 of the 12 modeled heteroclitic variants maintained conformational features comparable to those of their WT counterparts at the predicted HLA- and TCR-contact regions. Conversely, four variants displayed more pronounced alterations in the orientation of residues potentially interacting with the TCR and were therefore considered less suitable for supporting cross-recognition (**Figure 5**).

**Figure 5:**
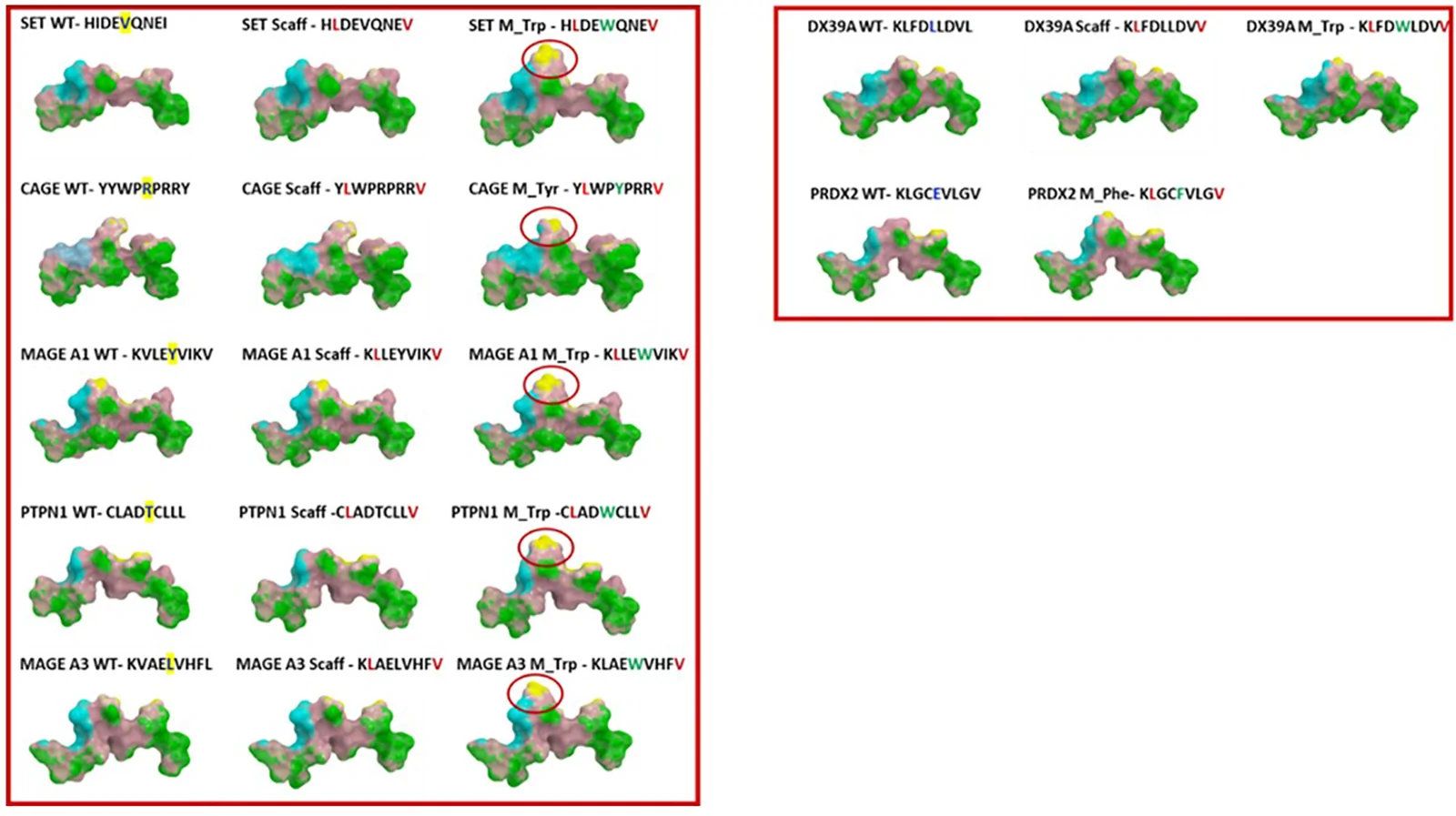
Structural modeling of wild-type, scaffold-HLA-A*02:01 and heteroclitic peptides. Three-dimensional models were generated using the HLA-A*02:01-peptide-TCR complex 1AO7 as the structural template. Representative models are shown for SET, CAGE, MAGE-A1, MAGE-A3, PTPN1, DDDX39A and PRDX2. Modified residues are highlighted in the peptide sequences, and circles indicate selected solvent-exposed P5 side chains.

The physicochemical properties of the substituted residues were also considered during peptide optimization. Conservative replacements preserving charge or polarity, as well as hydrophobic substitutions at anchor positions, were preferentially selected to maximize HLA binding while minimizing perturbation of the exposed peptide surface. Collectively, these structural analyses provided a comparative structural rationale for prioritizing variants showing preservation of the overall peptide backbone and TCR-accessible surface for subsequent experimental validation. These structural findings suggest that conservative, motif-guided substitutions can improve the predicted properties of the peptide HLA complex without necessarily disrupting the molecular surface recognized by WT-reactive T cells. The preservation of the relevant structural determinants therefore supported the selection of these variants for subsequent experimental assessment of T-cell cross-reactivity.

### 3.5 *In vitro* validation of HLA-A*02:01 binding and peptide–MHC complex stability

The binding capacity of the selected WT) and (HET peptide pairs was first evaluated in TAP-deficient T2 cells. Cells were incubated with increasing peptide concentrations ranging from 5 to 100 µM, and peptide-induced stabilization of cell-surface HLA-A*02:01 was quantified by flow cytometry as mean fluorescence intensity (MFI).

As shown in **Figure 6A**, most peptides induced a concentration-dependent increase in HLA-A*02:01 surface expression, although the magnitude of the response differed markedly among peptide pairs. Several HET variants consistently produced higher MFI values than their corresponding WT peptides across the tested concentration range. The most evident increases were observed for the SET-, CAGE- and BAG-derived variants, whereas PTPN1, MAGE-A1 and MAGE-A3 showed more moderate improvements. By contrast, PFETIN displayed only a minimal difference between WT and HET peptides, while the HET variants derived from DDX39A, PRAME and PRDX did not improve HLA-A*02:01 stabilization relative to their parental sequences.

**Figure 6:**
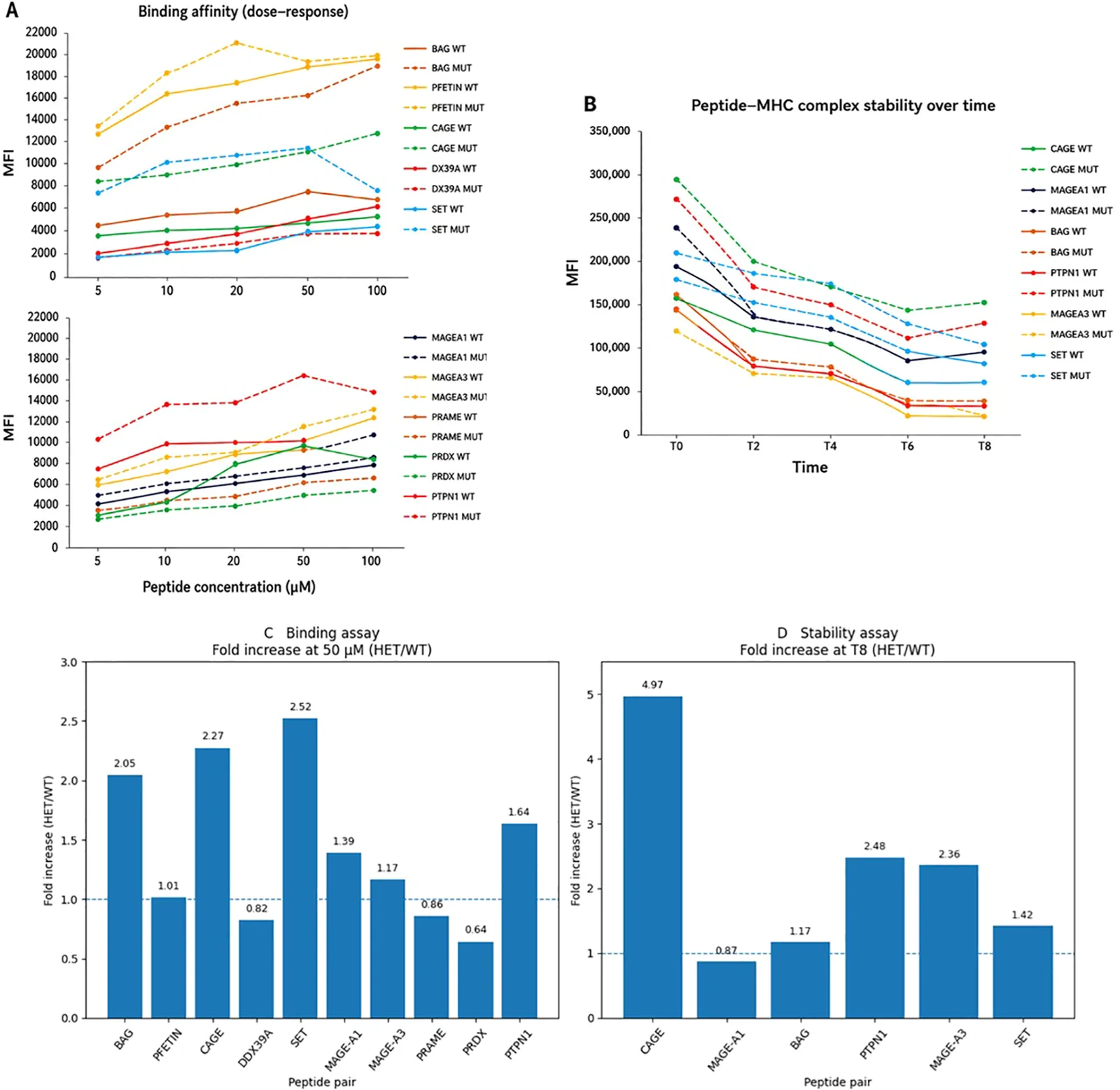
*In vitro* HLA-A*02:01 binding and peptide-MHC complex stability of wild-type and heteroclitic peptides. **(A)** CEM.T2 cells were incubated with increasing peptide concentrations (5-100 µM), and cell-surface HLA-A*02:01 expression was quantified as mean fluorescence intensity (MFI). Solid and dashed lines denote wild-type and heteroclitic peptides, respectively. **(B)** Following peptide loading and removal, HLA-A*02:01 expression was measured at 0, 2, 4, 6 and 8 h. Higher residual MFI indicates greater peptide-MHC complex stability. **(C)** HET/WT MFI ratio at 50 µM for each peptide pair in the binding assay. The dashed horizontal line at a ratio of 1 indicates equivalent HLA-A02:01 stabilization by HET and WT peptides; values >1 indicate greater stabilization by the HET peptide, whereas values <1 indicate lower stabilization relative to the corresponding WT peptide. **(D)** HET/WT MFI ratio at 8 h (T8) for the peptide pairs selected for the stability assay. The dashed horizontal line at a ratio of 1 indicates equivalent residual HLA-A02:01 expression for HET and WT peptides; values >1 indicate greater peptide–MHC complex stability for the HET peptide, whereas values <1 indicate lower stability relative to the corresponding WT peptide.

To provide a direct quantitative comparison, the HET/WT MFI ratio was calculated at 50 µM, a concentration at which most peptide pairs showed substantial HLA-A*02:01 stabilization without a clear additional gain at the highest dose tested. As shown in **Figure 6C**, the greatest fold increases were observed for SET, CAGE and BAG, with HET/WT ratios of 2.52, 2.27 and 2.05, respectively. PTPN1 also showed a clear improvement, with a 1.64-fold increase, followed by MAGE-A1 and MAGE-A3, with fold changes of 1.39 and 1.17. PFETIN showed essentially comparable binding between the two peptide forms, with a ratio of 1.01. Conversely, DDX39A, PRAME and PRDX displayed ratios below 1, indicating lower HLA-A*02:01 stabilization by the HET peptide at 50 µM.

Overall, the binding assay partially confirms the in silico prediction. In particular, SET, CAGE and BAG, which showed the largest predicted improvements in HLA-A*02:01 affinity, also displayed the strongest experimental increases in T2-cell surface stabilization.

Based on the binding results, the peptide pairs showing the most favorable HLA-A*02:01 stabilization profiles were selected for further analysis of peptide–MHC complex stability at different time points (T0 to T8). As shown in **Figures 6B**, HLA-A*02:01 expression generally decreased over time for all tested peptides, consistent with progressive peptide dissociation and turnover of surface peptide-MHC complexes. Nevertheless, the decay profiles differed substantially among peptide pairs. The CAGE HET peptide maintained markedly higher HLA-A*02:01 expression than the corresponding WT peptide throughout the observation period. Enhanced persistence was also observed for PTPN1 and MAGE-A3, whereas SET and BAG showed more modest differences between WT and HET complexes. In contrast, the MAGE-A1 HET peptide did not improve complex persistence relative to its WT counterpart.

The HET/WT MFI ratio calculated at T8 confirmed these observations (**Figure 6D**). CAGE showed the strongest increase in residual HLA-A*02:01 expression, with a 4.97-fold improvement over the WT peptide. PTPN1 and MAGE-A3 also displayed marked gains, with fold increases of 2.48 and 2.36, respectively. More limited improvements were observed for SET and BAG, with ratios of 1.42 and 1.17. By contrast, MAGE-A1 showed a ratio of 0.87, indicating no stability advantage for the HET variant at the final time point.

Taken together, these data demonstrate that heteroclitic optimization enhanced HLA-A*02:01 binding and/or peptide–MHC complex persistence for several sarcoma-associated epitopes. The binding analysis identified SET, CAGE and BAG as the strongest candidates, whereas the stability assay highlighted CAGE, PTPN1 and MAGE-A3.

Overall, the T2-cell experiments support a partial concordance between the in silico predictions and the *in vitro* validation and emphasize the need for direct experimental assessment of each optimized peptide.

### 3.6 Heteroclitic peptide priming induces functional T-cell responses cross-reactive with the corresponding wild-type epitopes

Cross-reactive T-cell responses between HLA-A*02:01-restricted heteroclitic peptides and their corresponding wild-type TAA-derived epitopes were evaluated ex vivo using PBMCs from HLA-A*02:01-positive sarcoma patients. To model the immune response potentially induced by vaccination with the optimized peptides, PBMCs were primed ex vivo with each HET peptide for 14 days. Cells were then re-stimulated in parallel with either the priming HET peptide or the corresponding WT epitope, and IFN-γ-producing cells were quantified by ELISpot.

As shown in **Figure 7**, PBMCs primed with HET peptides produced IFN-γ following restimulation with both the HET and corresponding WT sequences. Responses to WT restimulation demonstrate that HET-primed T-cell populations retained the ability to recognize antigenic determinants shared with the parental TAA-derived epitopes.

**Figure 7:**
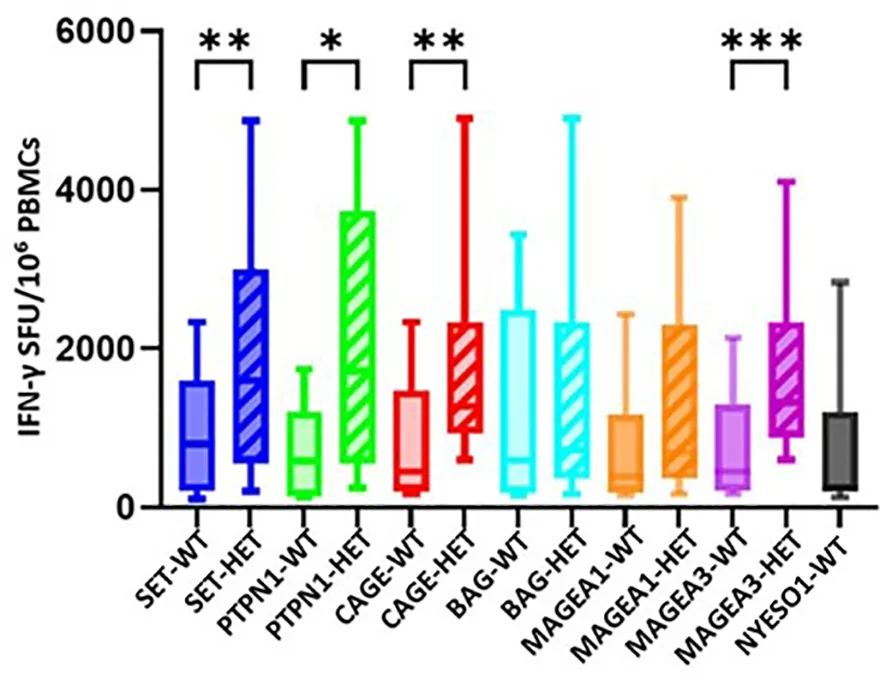
Heteroclitic peptide priming induces IFN-γ-producing T cells cross-reactive with the corresponding wild-type epitopes. PBMCs from HLA-A*02:01-positive sarcoma patients were primed ex vivo with individual heteroclitic peptides for 14 days and subsequently restimulated in parallel with either the priming heteroclitic peptide or the corresponding wild-type TAA-derived epitope. Antigen-specific responses were measured by IFN-γ ELISpot and expressed as background-subtracted spot-forming units per 10^6^ PBMCs. For NY-ESO-1, only the parental WT peptide was retained because heteroclitic optimization did not improve predicted HLA-A*02:01 binding.

Restimulation with HET peptides generally induced higher numbers of IFN-γ-producing cells than restimulation with the corresponding WT peptides. Significant increases were observed for SET (P < 0.01), PTPN1 (P < 0.05), CAGE (P < 0.01) and MAGE-A3 (P < 0.001). BAG and MAGE-A1 also showed higher median responses following HET restimulation, although the differences did not reach statistical significance. Considerable interpatient variability was observed across all peptide pairs, consistent with heterogeneity in the frequency and functional competence of peptide-reactive T-cell populations.

These findings indicate that heteroclitic peptide priming can enhance functional T-cell activation while preserving recognition of the corresponding WT epitopes. Thus, the optimized peptides act as more immunogenic stimuli without abolishing cross-recognition of the parental tumor-associated sequences.

### 3.7 Dextramer analysis identifies CD8^+^ T-cell populations cross-reactive with wild-type and heteroclitic epitopes

To determine whether CD8^+^ T cells expanded in response to the parental wild-type epitopes were also able to recognize the corresponding heteroclitic peptide–HLA complexes, PBMCs from HLA-A*02:01-positive sarcoma patients were stimulated *in vitro* with individual wild-type peptides for 7 days and subsequently analyzed by dual dextramer staining. For each peptide pair, cells were stained with HLA-A*02:01 dextramers loaded with either the wild-type peptide or the corresponding heteroclitic variant. The frequencies of wild-type dextramer-positive, heteroclitic dextramer-positive and double-positive CD8^+^ T cells were then determined within the CD3^+^CD8^+^ T-cell population.

As shown in **Figure 8**, wild-type dextramer-positive CD8^+^ T-cell populations were detected for all four peptide pairs analyzed, namely CAGE, SET, BAG and PTPN1. The highest mean frequency was observed for BAG, with 0.67 ± 0.46% wild-type dextramer-positive CD8^+^ T cells, followed by SET at 0.56 ± 0.68%, PTPN1 at 0.49 ± 0.40% and CAGE at 0.39 ± 0.25%. These findings confirmed the presence of circulating CD8^+^ T-cell populations capable of recognizing the parental HLA-A*02:01-restricted epitopes after short-term *in vitro* expansion.

**Figure 8:**
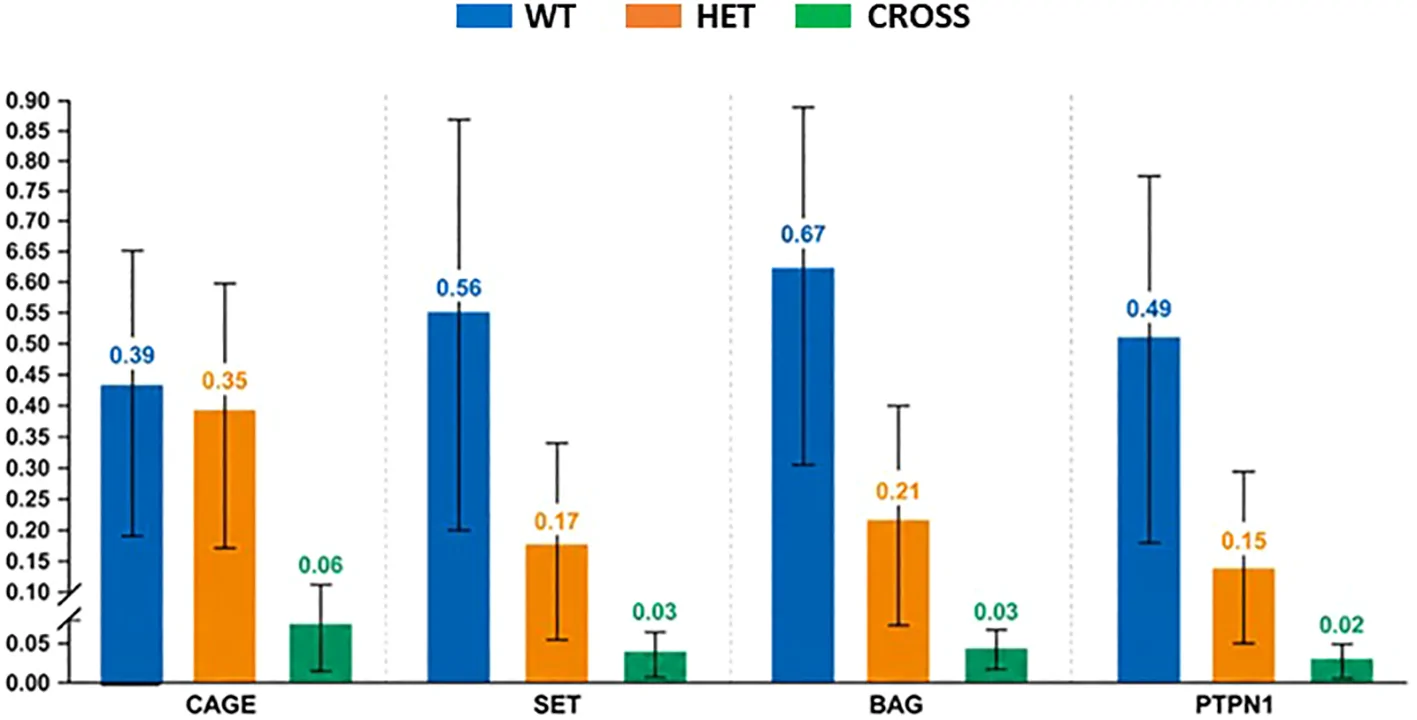
Wild-type-primed CD8^+^ T cells recognize the corresponding heteroclitic peptide–HLA-A*02:01 complexes. PBMCs from HLA-A*02:01-positive sarcoma patients were stimulated *in vitro* for 7 days with individual wild-type peptides derived from CAGE, SET, BAG or PTPN1. Cells were subsequently stained with HLA-A*02:01 dextramers loaded with either the wild-type peptide or the corresponding heteroclitic variant. Bars represent the percentages of CD3^+^CD8^+^ T cells positive for wild-type-loaded dextramers (WT, blue), heteroclitic-loaded dextramers (HET, orange) or both dextramers (CROSS, green). Data are shown as mean ± SD.

Importantly, wild-type-primed CD8^+^ T cells also bound dextramers loaded with the corresponding heteroclitic peptides. Heteroclitic dextramer-positive populations were detected at mean frequencies of 0.35 ± 0.24% for CAGE, 0.17 ± 0.15% for SET, 0.21 ± 0.16% for BAG and 0.15 ± 0.11% for PTPN1. Although these frequencies were consistently lower than those measured with the corresponding wild-type dextramers, their detection indicates that T cells expanded by the parental peptide can also recognize the optimized heteroclitic peptide–HLA complexes. The relatively high frequency observed for CAGE suggests particularly strong preservation of shared antigenic features between the parental and optimized peptide–HLA complexes.

Double-positive CD8^+^ T cells simultaneously binding both wild-type- and heteroclitic-loaded dextramers were also detected for all four peptide pairs. Mean frequencies were 0.06 ± 0.15% for CAGE, 0.03 ± 0.03% for SET, 0.03 ± 0.02% for BAG and 0.02 ± 0.02% for PTPN1. Although these populations represented only a small fraction of total CD8^+^ T cells, their reproducible detection supports the presence of T-cell populations capable of engaging both peptide–HLA complexes and is therefore consistent with cross-recognition of shared antigenic determinants.

The dextramer results complement the functional IFN-γ ELISpot data by addressing cross-reactivity from the opposite experimental direction. In the ELISpot assay, heteroclitic peptide priming generated T cells able to respond to the corresponding wild-type epitopes. In the dextramer assay, wild-type peptide priming expanded CD8^+^ T cells that also bound heteroclitic peptide–HLA complexes. Together, these reciprocal observations support the preservation of antigenic similarity between the optimized and parental epitopes.

Overall, these findings indicate that heteroclitic optimization did not abolish recognition by wild-type-reactive CD8^+^ T cells. This property is essential for the development of heteroclitic vaccine candidates, which must enhance peptide immunogenicity while maintaining antigenic determinants shared with the corresponding native tumor-associated epitopes.

## 4 Discussion

Peptide-based cancer vaccines provide a highly specific strategy for directing antitumor immunity, but their efficacy is often limited by the low immunogenicity of tumor-associated self-antigens (43). This limitation is particularly relevant in sarcomas, which are characterized by marked biological heterogeneity and a restricted number of validated shared antigenic targets. In this study, we developed an integrated strategy for the rational engineering of HLA-A*02:01-restricted heteroclitic peptides derived from sarcoma-associated proteins. The workflow combined motif-guided sequence optimization, in silico affinity prediction, structural modeling, T2-cell binding and stability assays, and ex vivo immunological validation in PBMCs from sarcoma patients.

The sequential design strategy consisted of optimization of the HLA-A*02:01 anchor residues at P2 and P9, followed by systematic substitution of the predominantly TCR-facing residue at P5. This approach generated marked predicted affinity improvements for several weakly binding wild-type epitopes. While the present strategy focused on the central TCR-facing P5 residue, additional TCR-facing positions may also contribute to peptide TCR interactions and could be explored individually or in combination in future studies, potentially allowing further refinement of the heteroclitic peptide design strategy.

The largest improvements were observed for CAGE, SET and BAG, whereas more modest improvements were obtained for peptides that already displayed favorable predicted binding. In contrast, no engineered NY-ESO-1 variant clearly outperformed the parental peptide, indicating that heteroclitic optimization is most useful when suboptimal HLA binding represents a major limitation and should not be applied indiscriminately.

The T2-cell experiments provided partial experimental support for the computational prioritization. At 50 µM, the SET-, CAGE- and BAG-derived heteroclitic peptides showed the largest increases in HLA-A*02:01 surface stabilization, with HET/WT fold changes of 2.52, 2.27 and 2.05, respectively. PTPN1, MAGE-A1 and MAGE-A3 displayed more moderate gains, whereas PFETIN showed comparable binding in its two forms and DDX39A, PRAME and PRDX did not improve after heteroclitic modification.

The incomplete concordance between predicted affinity and cellular binding is not unexpected, because peptide behavior in T2 cells is influenced by additional factors, including solubility, loading efficiency, exchange kinetics and the conformational stability of the peptide–MHC complex. These findings therefore support the use of computational prediction as a prioritization tool, but also emphasize the need for direct experimental validation.

Peptide-MHC stability provided complementary information. Among the peptide pairs selected on the basis of their binding profiles, CAGE showed the strongest increase in residual HLA-A*02:01 expression at T8, with an approximately 4.97-fold improvement. PTPN1 and MAGE-A3 also displayed marked gains, whereas BAG and SET showed more limited increases and MAGE-A1 did not benefit from heteroclitic modification. Thus, improved binding did not always translate into proportionally enhanced complex stability.

Structural modeling provided a complementary criterion for prioritizing heteroclitic variants by assessing whether the introduced substitutions were compatible with preservation of the overall peptide conformation and TCR-accessible surface. A common experimentally determined HLA-A*02:01–peptide–TCR framework was deliberately used to enable direct comparison of WT, scaffold and HET sequences under the same structural conditions. This approach was intended as a comparative structural assessment rather than a *de novo* prediction of peptide–HLA–TCR complexes or a quantitative estimate of binding affinity. While peptide-specific experimental structures or more recent structure-prediction approaches may provide additional information on sequence-dependent conformational differences, the present analysis allowed potentially disruptive variants to be distinguished from those preserving the overall structural features of the parental epitopes. Importantly, because structural similarity alone cannot establish biological cross-recognition, this prediction was subsequently evaluated experimentally by ELISpot and dextramer analyses. The *ex vivo* ELISpot experiments showed that PBMCs primed with heteoclitic peptides responded not only to restimulation with the optimized peptide but also to the corresponding WT epitope. Restimulation with HET peptides generally elicited stronger IFN-γ responses, with significant increases for SET, PTPN1, CAGE and MAGE-A3. Importantly, the detection of responses after WT restimulation indicates that HET priming expanded T-cell populations capable of recognizing antigenic determinants shared with the parental TAA-derived sequence.

The dextramer analysis provided complementary evidence of cross-recognition from the reciprocal experimental direction. WT-primed CD8^+^ T cells bound not only WT-loaded dextramers but also dextramers carrying the corresponding heteroclitic peptides. Double-positive populations capable of binding both reagents were detected for CAGE, SET, BAG and PTPN1. Although these populations were small, their reproducible detection supports preservation of shared antigenic features between the parental and optimized peptide–HLA complexes.

Taken together, the ELISpot and dextramer results address a central requirement for heteroclitic vaccine design. An optimized peptide must be more immunogenic than its parental counterpart while retaining sufficient antigenic similarity to induce or engage T cells relevant to the native tumor-associated epitope. The reciprocal cross-recognition observed here suggests that this balance was achieved for several of the selected candidates.

The lack of complete agreement among in silico affinity, T2-cell binding, peptide–MHC stability and ex vivo immunogenicity also highlights the complexity of peptide selection. CAGE showed the most consistent performance across all levels of analysis, whereas PTPN1 and MAGE-A3 displayed particularly strong stability and functional responses despite more moderate predicted or binding improvements. SET and BAG showed substantial predicted and experimental binding gains, but more limited stability advantages. These differences demonstrate that no single parameter is sufficient to identify the most promising vaccine candidate and support the value of an integrated, multidimensional selection strategy. By integrating HLA binding prediction, structural conservation, cellular stabilization, peptide-MHC persistence and reciprocal T-cell cross-recognition, this approach identifies candidates that are both more immunogenic and biologically connected to their parental tumor-associated epitopes.

An additional consideration in the development of heteroclitic peptides concerns the potential biological consequences of markedly increasing peptide–HLA binding and complex stability. While enhanced and prolonged peptide presentation may improve the recruitment and activation of antigen-specific T cells, a substantial increase in peptide–HLA stability could potentially modify the threshold for T-cell activation and broaden the repertoire of responding T-cell clonotypes. In principle, this may increase the possibility of unintended cross-reactivity or activation of lower-affinity T-cell populations *in vivo*. Thus, optimization of heteroclitic peptides should not be aimed solely at maximizing HLA binding, but should maintain an appropriate balance between peptide–MHC stability, immunogenicity and antigen specificity. In our study, reciprocal recognition of WT and HET peptide–HLA complexes supports preservation of the antigenic relationship between the optimized and parental epitopes. Nevertheless, these ex vivo observations cannot exclude additional off-target or bystander responses that may emerge *in vivo*. Further preclinical development of the most promising candidates should therefore include dedicated assessment of T-cell specificity, cross-reactivity and safety before their potential incorporation into therapeutic vaccine formulations.

In summary, our study shows that rational heteroclitic engineering can enhance the immunological properties of sarcoma-associated epitopes while preserving recognition of the corresponding WT antigens. These findings provide a mechanistic and translational rationale for the further development of heteroclitic peptide-based immunotherapies in sarcoma and potentially in other low-immunogenicity tumors.

## 5 Conclusions

We developed a sequential HLA-A*02:01-guided strategy for the engineering of heteroclitic peptides derived from sarcoma-associated proteins. Anchor optimization followed by controlled substitution of the TCR-facing P5 residue generated variants with improved predicted affinity, enhanced HLA-A*02:01 stabilization and, in selected cases, prolonged peptide-MHC complex stability.

*Ex vivo* analyses showed that heteroclitic peptide priming induced functional T-cell responses that remained cross-reactive with the corresponding WT epitopes. Reciprocal dextramer staining further demonstrated that WT primed CD8^+^ T cells could recognize heteroclitic peptide–HLA complexes.

Overall, these findings indicate that rational heteroclitic engineering can enhance the immunogenicity of selected sarcoma-associated epitopes while preserving antigenic determinants shared with the parental sequences. This integrated approach provides a promising framework for the development of multi-epitope therapeutic vaccines for sarcoma and supports further validation in tumor-recognition and cytotoxicity models.

## Data Availability

The original contributions presented in the study are included in the article/supplementary material, further inquiries can be directed to the corresponding author/s. The raw data are available at: https://doi.org/10.5281/zenodo.22793878.

## Supplementary material

Supplementary Figure 1 Representative flow-cytometry gating strategy for dextramer analysis. Representative gating strategy used to identify antigen-specific CD8^+^ T-cell populations. Lymphocytes were sequentially gated, followed by identification of CD3^+^CD8^+^ T cells. WT- and HET-dextramer staining was subsequently used to identify WT-positive, HET-positive and WT/HET double-positive CD8^+^ T-cell populations.

Supplementary Table 1 HLA typing was specifically performed for HLA-A02:01, as all candidate epitopes investigated in this study were selected and experimentally validated in the context of HLA-A02:01 restriction.
