# Supplementary material for: Peptide optimization to improve antigen presentation and antitumor responses in sarcoma
Source: Front Immunol. 2026 Oct 1;17:1960095. doi: 10.3389/fimmu.2026.1960095 (PMC13669185; doi:10.3389/fimmu.2026.1960095)
Supplement: Supplementary file 1 [file Table1.docx]

| **Patient cohort** | **Number** | **% of total cohort** |
| --- | --- | --- |
| **Total sarcoma patients HLA-typed** | **70** | **100.0** |
| HLA-A*02:01 positive | 22 | 31.4 |
| HLA-A*02:01 negative | 48 | 68.6 |
