# Supplementary material for: Peptide optimization to improve antigen presentation and antitumor responses in sarcoma
Source: Front Immunol. 2026 Oct 1;17:1960095. doi: 10.3389/fimmu.2026.1960095 (PMC13669185; doi:10.3389/fimmu.2026.1960095)
Supplement: Supplementary file 2 [file Table2.docx]

**Table 1: Paired sarcoma peptides selected**

| **Name** | **SEQ** |
| --- | --- |
| NY-ESO-1 -WT | **SLLMWITQC** |
| PRDX5-WT | **LLDDSLVSI** |
| PRDX5-HET | **LLDDWLVSV** |
| CAGE-WT | **YYWPRPRRY** |
| CAGE-HET | **YLWPYPRRV** |
| MAGEA1-WT | **KVLEYVIKV** |
| MAGEA1-HET | **KLLEYVIKV** |
| MAGEA3-WT | **KVAELVHFL** |
| MAGEA3-HET | **KLAELVHFV** |
| PTPN1 -WT | **CLADTCLLL** |
| PTPN1-HET | **CLADWCLLV** |
| SET -WT | **HIDEVQNEI** |
| SET-HET | **HLDEWQNEV** |
| PRDX2 -WT | **KLGCEVLGV** |
| PRDX2-HET | **KLGCFVLGV** |
| PRAME -WT | **NLTHVLYPV** |
| PRAME-HET | **NLTHWLYPV** |
| DX39A-WT | **KLFDLLDVL** |
| DX39A-HET | **KLFDWLDVV** |
| PFETIN-WT | **SLGDELLPL** |
| PFETIN-HET | **SLGDWLLPV** |
| BAG-WT | **YMSKVLDGV** |
| BAG-HET | **YLSKWLDGV** |
